# Intervals of endovascular treatment for coincidental non-adjacent unruptured aneurysms in patients with symptomatic intracranial atherosclerotic stenosis

**DOI:** 10.3389/fneur.2022.1004536

**Published:** 2022-09-23

**Authors:** Hengwei Jin, Jing Wang, Xiangyu Meng, Youxiang Li, Hongwei He

**Affiliations:** ^1^Department of Interventional Neuroradiology, Beijing Neurosurgical Institute, Beijing Tiantan Hospital, Capital Medical University, Beijing, China; ^2^Department of Neurology, The 4th Hospital of Handan, Handan, China; ^3^Neurosurgery Department, The First Hospital of Hebei Medical University, Shijiazhuang, China

**Keywords:** endovascular treatment, intracranial atherosclerotic stenosis (ICAS), intracranial aneurysm, interval, safety

## Abstract

**Background and purpose:**

To explore the safety of endovascular therapy for concomitant non-adjacent unruptured intracranial aneurysms (UIAs) which is incidentally found in severe patients with symptomatic intracranial atherosclerotic stenosis at the same session and different sessions.

**Methods:**

Patients between January 2019 to December 2020 were retrospectively reviewed at our institution. Patients with concomitant non-adjacent incidental UIA in severe symptomatic intracranial atherosclerotic stenosis, who underwent endovascular treatment for both lesions were included. They were divided into two groups according to the intervals (The aneurysm was treated at the same session as stenosis or at separated sessions). The demographics, procedure details, complications, and clinical outcomes were compared between groups.

**Results:**

A total of 22 patients were involved. In total, ten patients underwent endovascular treatment for UIA and stenosis at one session and 12 patients at separate sessions. In total, three (13.6%) patients experienced procedural related complications, including 2 (20%) in the one session group and 1(8.3%) in the separate sessions group. Follow-up (Range 6–12, mean = 8.5 months) results showed good clinical outcome in all the patients. There is no statistical significance in terms of complication rate and unfavorable clinical outcome between groups.

**Conclusions:**

Non-adjacent concomitant UIA and severe symptomatic intracranial atherosclerotic stenosis will not pose additional endovascular treatment risks. Both simultaneous endovascular management and short intervals between separated procedures are technically feasible and safe.

## Background

Concomitant existence of cerebral atherosclerotic stenosis and incidental unruptured intracranial aneurysms (UIAs) are supposed to have high-treatment risks (1). Quandaries are mainly about the location and treatment intervals of concomitant lesions. If the UIA was managed simultaneously with the stenosis, embolization of the UIA may lengthen the procedure, which may increase the possibility of ischemic events. Conversely, if the UIA was managed in separate stage, the angioplasty of the stenosis may cause UIA rupture due to hemodynamic changes such as increased blood flow and pressure. No guidelines currently exist to guide practitioners whether one procedure or separate procedures should be performed when both the lesions require management. The risk of ischemic stroke and the risk of UIA rupture must be evaluated and balanced before treatment (2–4). A few studies have reported patients with concomitant extracranial carotid stenosis and UIA, and also patients with adjacent atherosclerotic stenosis and UIA. The overall complication rate is not high and clinical outcomes turn out to be good (5–12). To the best of our knowledge, few authors have reported the safety of endovascular treatment for non-adjacent concomitant intracranial symptomatic atherosclerotic stenosis and UIA. In the current study, we evaluated the safety of endovascular treatment for non-adjacent incidental UIA in symptomatic severe intracranial atherosclerotic stenosis patients in the same session and separate sessions.

## Materials and methods

### Patients

This study was approved by the ethics committee of our hospital, and all patients provided written informed consent. We retrospectively reviewed patients from January 2019 to December 2020 at our institution. Inclusion criteria are: patients with symptomatic (TIA or/and stroke) severe intracranial atherosclerotic stenosis and incidental non-adjacent unruptured intracranial aneurysm. Severe is defined as 70%−99% degree of stenosis based on the Warfarin–Aspirin Symptomatic Intracranial Disease Study (WASID) criteria (13, 14). The symptoms of patients were all attributed to severe intracranial stenosis, and the UIA was asymptomatic and incidentally found; Both the stenosis and the aneurysm are managed by endovascular therapy. The treatment of the aneurysm is following that of the stenosis. Patients' demographics, clinical information, procedure details, complications, and clinical follow-up results were collected. Patients were excluded if the initial modified Rankin Scale (mRS)≥3; Patients were also excluded if there was no complete information or lost to follow-up. Missing information include but not confined to image materials (such as detailed characteristics of stenosis and aneurysm), treatment details (such as method and materials) and complications (such as symptoms and prognosis). Patients were categorized into one-session group if the stenosis and the aneurysm were managed simultaneously, and categorized into separate-session group if not. Subgroup analysis were also performed between patients with stenosis and aneurysm located ipsilaterally or non-ipsilaterally. The demographics, complications, and clinical outcomes were compared between groups.

### Definitions of variables

Patients' demographics and clinical data were collected. Initial clinical presentations were ischemic symptoms directly resulted by relative stenosis, including numbness of anybody parts, weakness of limbs, vertigo and slurred speech, etc. The UIAs were incidentally found when performing medical examination due to ischemic symptoms. “Adjacent” is defined as the presence of both the stenosis and the aneurysm in the same arterial anatomic segment. Otherwise, they are non-adjacent. In the subgroup analysis, the relationship between UIA and stenosis is categorized into two types according to the side: ipsilateral and non-ipsilateral. The former refers to both UIA and stenosis located at left CCA system (left ICA, left MCA, and left ACA), right CCA system (right ICA, right MCA, and right ACA) or posterior circulation (unless UIA and stenosis located at bilateral Vas, respectively).

Periprocedural complications are categorized into ischemic and hemorrhagic types. Ischemic complication is defined as any additional neurologic deficits compared with pre-operation and infarctions confirmed by CT/MRI within 30 days after procedure (15). Hemorrhagic complication is defined as intracranial hemorrhage (ICH/SAH) happened within 7 days after procedure confirmed by CT (16). All the patients were evaluated with the mRS before procedure and at last follow-up. An mRS 0–2 (independent) is regarded as favorable clinical outcome and mRS≥3 (dependent) is regarded as unfavorable clinical outcome.

### Stenosis angioplasty and UIA embolization

Treatment indication for stenosis and UIA is strictly according to the Guidelines from the American Heart Association/American Stroke Association (AHA/ASA), respectively (17, 18). There is no guideline concerning treatment intervals. The general principle of one-session treatment at our center includes the following: (1) The aneurysm is of high rupture risk (such as daughter aneurysm, unregular shape, and relatively big sized, etc.). (2) The aneurysm is located ipsilaterally with stenosis since management of stenosis will lead to high blood pressure and rupture risk of the aneurysm. The aforementioned principles are combined with individual characteristics (such as age and general health condition) and patient's will. Dual antiplatelet therapy that comprised aspirin (100 mg/d) and clopidogrel (75 mg/d) was initiated at least 5 d before stent implantation. All the procedures were performed under general anesthesia. During the intervention, 3,000–4,000 IU of heparin was administered, and additional 1,000 IU per h. A 6- to 8-F sheath was inserted through the femoral artery and a 6- to 8-F guiding catheter was navigated into the internal carotid or the vertebral artery. The guiding catheter was flushed *via* a pressure bag with saline containing 3,000 U of heparin/500 ml. For angioplasty, balloon dilation with or without stent was performed according to the standardized routine form AHA/ASA (18) and our institution. The UIA was embolized with coils alone, stent-assisted coils, or pipeline according to standardized routine of our institution. Before and immediately after the procedure, the neurological function of every patient was evaluated. All the patients received in-person or telephone follow-up at least 6 months after operation. The final mRS score was based on their functional status at last follow-up.

### Statistical analyses

Patients' characteristics were described with frequencies for categorical variables and mean standard deviation for continuous variables. Categorical variables were compared using Fisher exact test or the Pearson χ^2^ test. Continuous variables were compared between groups using student's *t*-test. All the *P* values were reported as two-sided. *P* < 0.05 was considered significant. All the statistical analyses were conducted using SPSS 22.0 (Chicago, IL, USA).

## Results

### Patients' demographics and clinical information

A total of 22 patients meets the inclusion criteria and involved in the study. Age ranges from 40 to 82 years old (mean ± SD: 61.1±7.6 years old). In total, twelve stenosis located at anterior circulation (intracranial segment of ICA or MCA) and 10 stenosis at posterior circulation (intracranial segment of VA or BA). In total, seven patients presented as TIA and another 15 patients presented as ischemic stroke, which is confirmed by DWI. The distribution of infarctions is consistent with that of severe stenosis. In anterior circulation, the infarction mainly located at cortex, basal ganglia, periventricular area and centrum semiovale. In the posterior circulation, the infarction mainly located cerebellar hemisphere and brain stem. The initial mRS was 0 in 16 patients, 1 in 4 patients, and 2 in 2 patients. In total, fifteen aneurysms located at anterior circulation and 7 located at posterior circulation. Including 9 at ICA, 3 at anterior communicating artery, 2 at MCA, 6 at VA, 1 at BA, and 1 at anterior artery. The maximum diameter of UIA range from 2 to 8 mm (mean ± SD: 4.2±1.3 mm). in total, twelve patients have stenosis and aneurysms located ipsilaterally, and other 10 patients have lesions located non-ipsilaterally. Patients' demographics and clinical information are demonstrated in Table 1. The demographics and clinical information of one session and separate session patients compared (Table 2).

**Table 1:** Details of demographics, stenosis, and aneurysm of patients.

**Patient No**.	**Group**	**Age/** **gender**	**L. of stenosis**	**T. of stenosis**	**L. of aneurysm**	**Morphology/** **Max. size(mm)**	**T. of aneurysm**	**Complications**	**Increased mRS**
1	One S.	64/F	L M1	Balloon	L C7*	Sac. /5	C+S	None	0
2	One S.	61/M	R C6	B+S	R MCA*	Sac. /4	Coil	None	0
3	One S.	64/F	R V4	B+S	BA*	Dis. /3	C+S	None	0
4	One S.	49/F	BA	Balloon	ACoM	Sac. /3	C+S	None	0
5	One S.	71/F	BA	B+S	L V4*	Dis. /4	C+S	None	0
6	One S.	53/M	R MCA	Balloon	R C5*	Sac. /3	C+S	Yes	0
7	One S.	63/M	R V4	Balloon	L V4	Sac. /5	C+S	None	0
8	One S.	66/F	L MCA	Balloon	L C6*	Sac. /6	FD	None	0
9	One S.	61/M	BA	B+S	R V4*	Dis. /6	C+S	None	0
10	One S.	57/F	L MCA	Balloon	L C7*	Sac. /3	C+S	Yes	1
11	S.S.	61/M	R C6	B+S	L V4	Dis. /8	FD	Yes	1
12	S.S.	65/M	L MCA	Balloon	L C5*	Sac. /3	C+S	None	0
13	S.S.	53/F	L V4	Balloon	R A1	Sac. /2	Coil	None	0
14	S.S.	56/M	R V4	B+S	ACoM	Sac. /4	C+S	None	0
15	S.S.	60/M	L MCA	B+S	R C6	Sac. /3	C+S	None	0
16	S.S.	82/M	BA	Balloon	ACoM	Sac. /4	Coil	None	0
17	S.S.	61/M	L C7	B+S	R V4	Dis. /4	FD	None	0
18	S.S.	70/M	R MCA	B+S	L C6	Sac. /3	Coil	None	0
19	S.S.	56/F	BA	Balloon	L V4*	Sac. /4	C+S	None	0
20	S.S.	58/M	L MCA	B+S	L C6*	Sac. /6	FD	None	0
21	S.S.	48/M	R V4	Balloon	R C5	Sac. /4	Coil	None	0
22	S.S.	66/M	R C6	B+S	R MCA*	Sac. /5	FD	None	0

One S., One session; S.S., Separate sessions; L. of stenosis, Location of stenosis; T. of stenosis, Treatment of stenosis; L. of Aneurysm, Location of Aneurysm; Sac., Saccular aneurysm; Dis., Dissection aneurysm; T. of aneurysm, Treatment of aneurysm; B+S, Balloon and Stent; C+S, Coils and stent; M, Male; F, Female;

* Aneurysm is ipsilateral located with stenosis; FD, Flow diverter.

**Table 2:** Comparison of demographics and clinical information between groups.

**Parameters**	**Total**	**One session**	**Separate sessions**	***P*-value**
Total patients, n (%)	22	10	12	-
Age (years), mean(±SD)	61.1 ± 7.6	60.9 ± 6.4	61.3 ± 8.8	0.898
Gender, male, *n* (%)	14 (63.6)	4 (40)	10 (83.3)	0.074
Presented as stroke, *n* (%)	15 (68.2)	7 (70)	8 (66.7)	0.867
Hypertension, *n* (%)	18 (81.8)	7 (70)	11 (91.7)	0.293
Diabetes, *n* (%)	11 (50)	5 (50)	6 (50)	1.000
Location of aneurysm, n (%)				0.452
Anterior circulation	15 (68)	6 (60)	9 (75)	
Posterior circulation	7 (32)	4 (40)	3 (25)	
Size of UIAs(mm), Mean(±SD)	4.1 ± 1.5	4.1 ± 1.4	4.2 ± 1.6	0.918
Location of stenosis, *n* (%)				0.696
Anterior circulation	12 (54)	5 (50)	7 (58)	
Posterior circulation	10 (46)	5 (50)	5 (42)	

SD, standard deviation; UIA, unruptured intracranial aneurysm.

### Treatment

**Figure 1:**
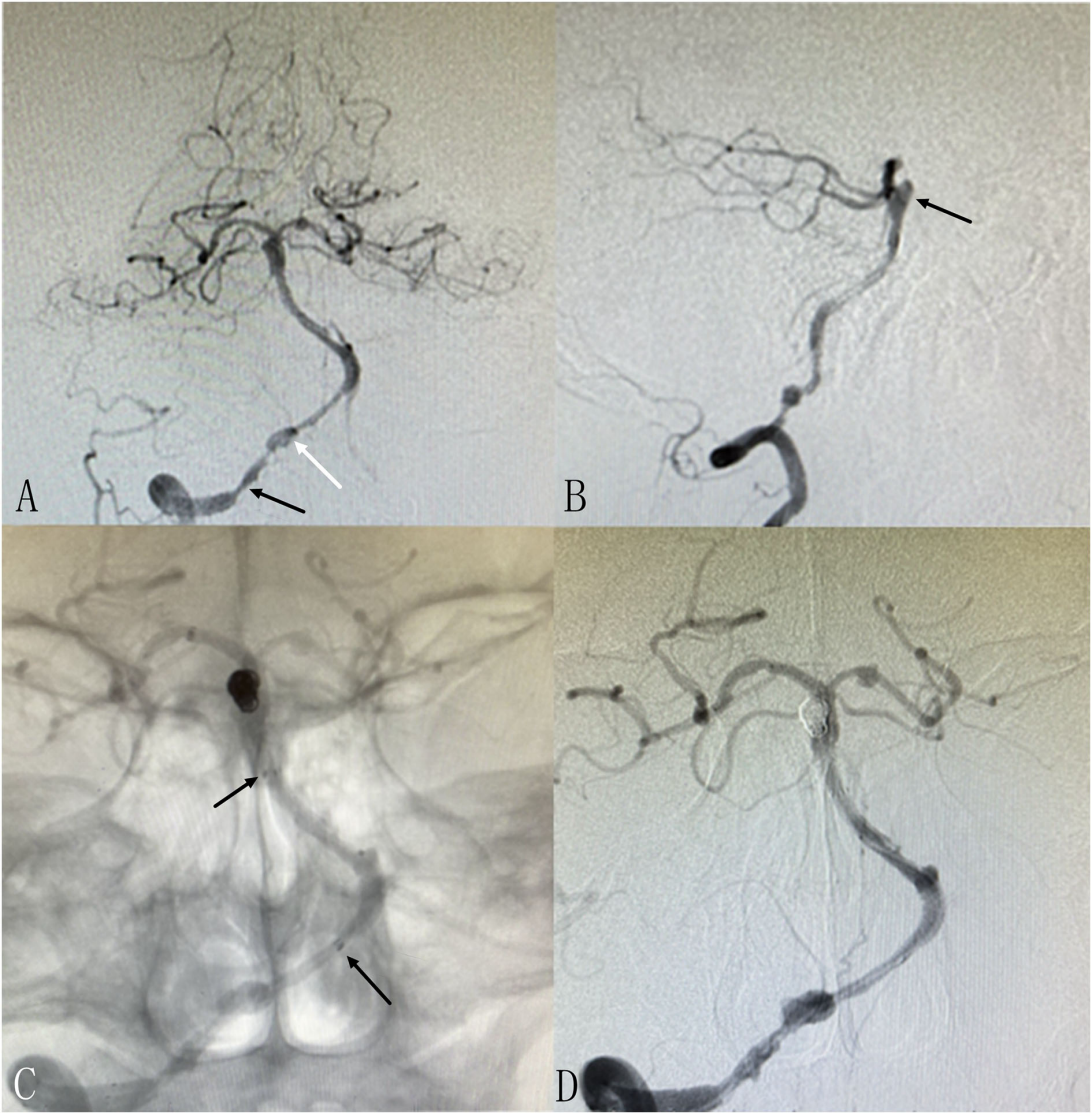
Patient 3: Single session angioplasty of stenosis and UIA embolization. The patient had mild blurred vision for 10 days. DSA revealed severe stenosis at intracranial segment of the right vertebral artery as well as an basilar aneurysm [**(A,B)**, black arrows]. Balloon dilatation and stenting was performed successfully for the stenosis. The aneurysm was embolized with coils and stent in in the same session **(C,D)**. The markers of the stents are marked [**(C)**, black arrows]. There is another small aneurysm located at V4 of RVA adjacent to the stenosis [**(A)**, white arrow], which is small in size and regular in shape and decided to be observed temporarily. Another procedure will be performed if needed.

**Figure 2:**
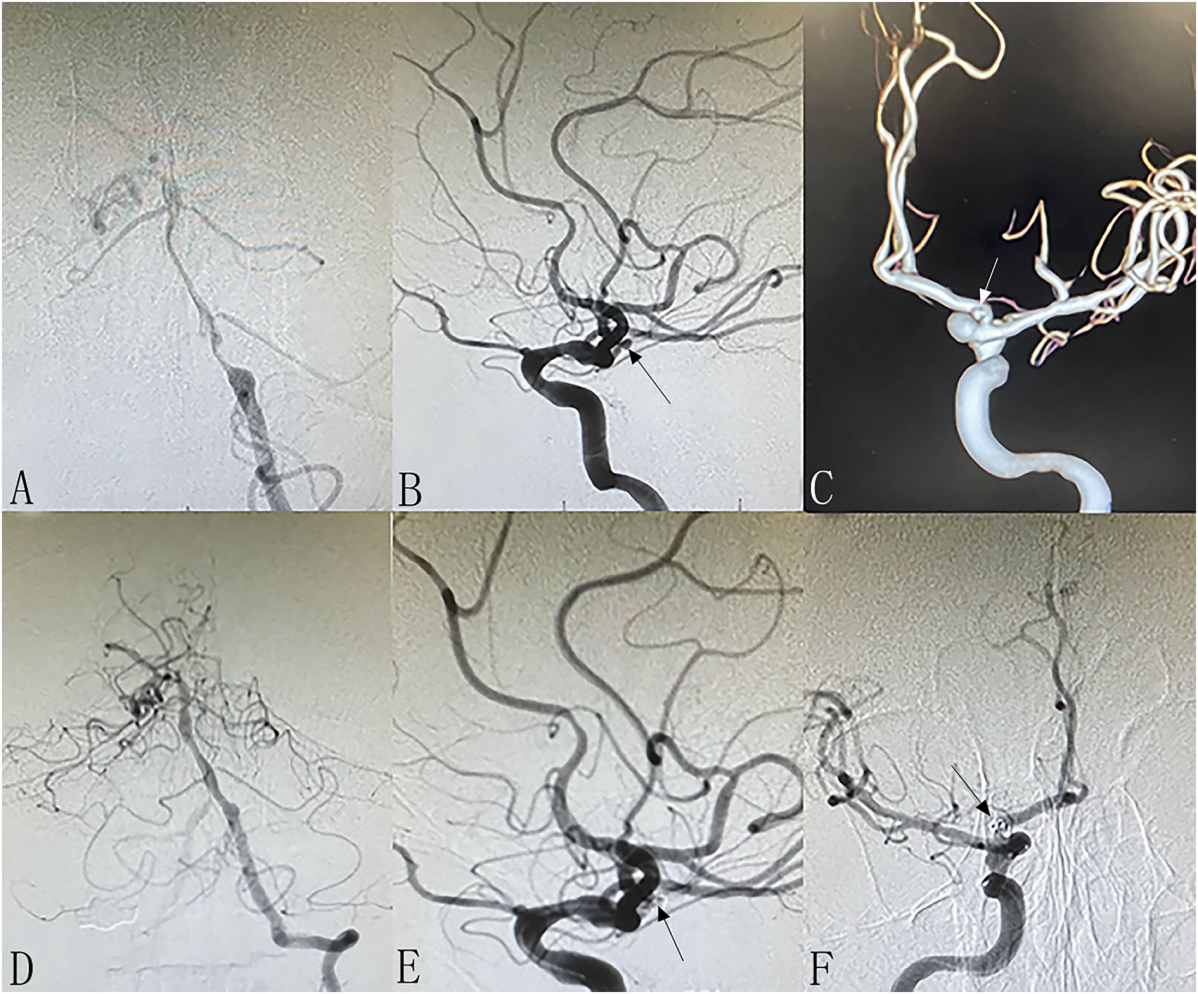
Patient 13: Separated sessions angioplasty of stenosis and UIA embolization. The patient had vertigo for 2 weeks. DSA showed severe stenosis at left V4 **(A)** and non-ipsilateral small right anterior artery aneurysm [**(B,C)**, arrows]. In the first procedure, we performed balloon dilation and stent implantation for the stenosis **(D)**. One week later, we performed coiling of the aneurysm successfully **(E,F)**.

**Figure 3:**
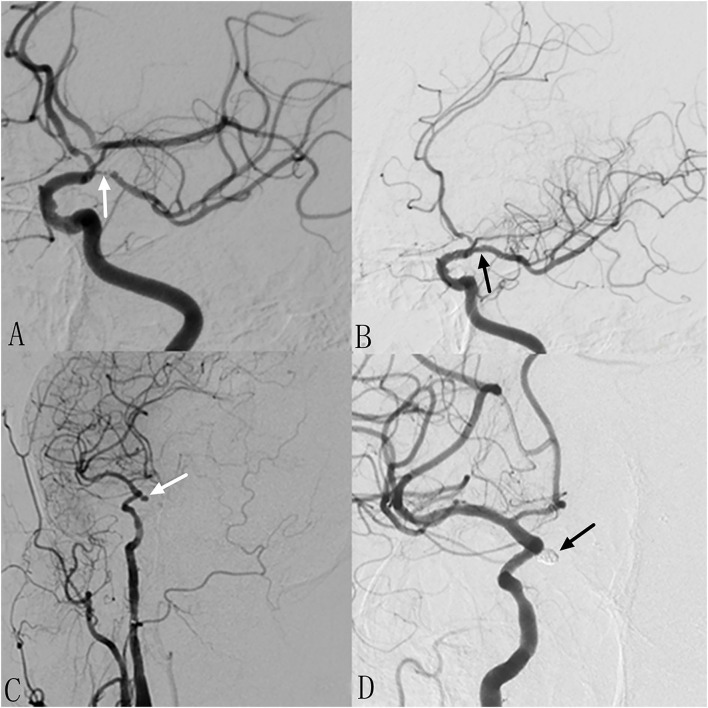
Patient 15: Separated sessions angioplasty of stenosis and UIA embolization. The patient had weakness in the right limbs for 1 month. DSA showed severe stenosis at left MCA **(A)** and non-ipsilateral post communicating artery aneurysm **(C)**. In the first procedure, we performed balloon dilation and stent implantation for the stenosis **(B)**. One month later, we performed stent assisted coiling of the aneurysm successfully **(D)**.

A total of 34 procedures were performed, including 10 procedures for patients underwent endovascular treatment for stenosis and aneurysm at the same session (Figure 1) and 24 procedures for patients at separate sessions (Figures 2, 3). In separate session group, the interval ranges from 1 week to 1 month. The surgical interventions for the same patient were performed by the same operator. A total of 22 stenosis was treated, including 11 (50.0%) balloon dilation and 11(50.0%) balloon dilation followed by stenting. A total of 22 UIAs were treated, including coil embolization of 5 (22.7%) patients, stent assistant coil embolization of 12 (54.5%) patients, and flow diverter of 5 (22.7%). Compared with patients who have non-ipsilateral lesions, patients with ipsilateral lesions are more likely to be treated in one session (80 vs. 33.3%, *p* = 0.043) (Table 3).

**Table 3:** Comparison of treatment details between groups.

**Parameters**	**One session**	**Separate sessions**	***P* value**
No. of cases, *n* (%)	10 (%)	12 (%)	-
Aneurysm, *n* (%)			0.091
Coiling	1 (10)	4 (33.3)	
Stent assisted coiling	8 (80)	4 (33.3)	
Pipeline	1 (10)	4 (33.3)	
Stenosis, *n* (%)			0.670
Balloon dilation	6 (60)	5 (41.7)	
Balloon dilation and stent	4 (40)	7 (58.3)	
Ipsilateral located, (%)	8 (80)	4 (33.3)	0.043
Complications, *n* (%)	2 (20%)	1 (8.3%)	0.571

### Complications and clinical follow-up

A total of 3 (13.6% of patients and 8.8% of procedures) procedural related complications happened and all of them are ischemic types. In total, one patient (patient 6) underwent RMCA stenosis angioplasty and RICA aneurysm embolization at the same session. He experienced transient contralateral limb dysfunction and completely recovered at discharge. In total, one patient (patient 10) received LMCA stenosis angioplasty and LICA aneurysm embolization at one session. He suffered contralateral limb dysfunction. MRI confirmed small infarctions which is considered caused by perforators occlusion or microthrombus. He has an mRS of 1 at last follow-up. The other patient (patient 11) is form separate session group. He experienced parent artery occlusion after pipeline implantation for left vertebral artery aneurysm. He has an mRS of 1 at last follow-up. In this cohort, follow-up period ranges from 6 to 12 months (mean 8.5 months). There is no statistical difference in terms of complication rate (20 vs. 8.3%, *p* = 0.571) and clinical outcome between one-session group and separate-session group. Subgroup analysis showed that there is also no statistical significance in terms of complication rate and clinical outcome regarding non-ipsilateral or ipsilateral location of stenosis and aneurysm (*p* = 1.000). No hemorrhagic complication happened during intervals in separate session patients. in total, two patients (patients 10 and 11) had an increased mRS of 1. All the patients achieved favorable clinical outcome (mRS < 3). Treatment details and follow-up results are listed in Tables 1, 3.

## Discussion

In total, one dilemma tangling us is whether we should manage the stenosis and the UIA in one procedure or in separate procedures when we meet patient with symptomatic intracranial atherosclerotic stenosis and incidental non-adjacent UIA. No consensus has been achieved to guide endovascular treatment of concomitant symptomatic intracranial atherosclerotic stenosis and UIA. Angioplasty may increase the risk of UIA rupture from altered hemodynamics, especially when they are located ipsilaterally. Embolization of an UIA immediately after managing stenosis pose additional ischemic risks. We must evaluate and balance the additional risks of UIA rupture from altered hemodynamics and ischemic risks from extended procedure time. In this study, we reviewed consecutive patients in a single center for patients with concomitant symptomatic intracranial atherosclerotic stenosis and incidental non-adjacent UIA, focusing on the treatment intervals and complications. A total of 22 patients underwent endovascular therapy for the stenosis and UIA were involved. For all patients, the stenosis was firstly managed since they are the responsible lesions, followed by UIA embolization during the same or another procedure. The complication rate of one session group is not significantly higher than separate session group, indicating that one session endovascular management for both lesions do not pose additional risks. A single-stage endovascular procedure is a recommendable choice to treat both lesions. Compared with that of a multiple-stage procedure, the outcome of single-stage endovascular treatment showed no significant difference in neither complications nor prognosis. A single-stage procedure could not only eliminate the need for an additional admission but also eliminate the further cost and discomfort of the patients (12).

One of the relationships of non-adjacent stenosis and UIA is ipsilateral and non-ipsilateral location. It is one of the important factors we should keep in mind when making operation plans. When they are ipsilaterally located, the management of stenosis will have direct influence on the aneurysm. The correction of a severe stenosis may increase the blood and pressure of the UIA, and then increase the chance of enlargement and rupture. For these reasons, one session approach was recommended in most of the ipsilateral cases. In the current study, 12 patients have stenosis and UIA located ipsilaterally and 8 (66.7%) were managed in one session. Ni et al. reported a series of single-stage endovascular treatment for severe cranial artery stenosis coexisted with ipsilateral distal tandem aneurysm in 10 patients. They compared their patients with previous literatures and concluded that single-stage endovascular procedure is feasible and effective for the lesions (12). When they are non-ipsilaterally located, the hemodynamic influence on the UIA is relatively smaller when angioplasty is performed for the stenosis, while there are other concerns such as ischemic risks resulted by prolonged procedure time and rupture risks during intervals.

The treatment of adjacent and non-adjacent lesions is different. When stenosis and UIA located adjacently, the angioplasty of symptomatic stenosis will inevitably disturb the UIA, and in most circumstances both lesions are within the coverage area of the balloon or stent because they are very near to each other. This may be associated with a greater risk of complications and the UIA is usually managed simultaneously to decrease the risks as much as possible. Moreover, adjacent lesions also bring difficulties to balloon dilation or stent placement because of the need to manage an aneurysm. Although stent placement can protect the neck of the aneurysm, dilation of the stenotic vessel can increase the risk of aneurysm rupture (19). When stenosis and UIA located non-adjacently, they could be managed separately since there is adequate distance between lesions. The UIA will not be directly disturbed when managing stenosis. The biggest influence after angioplasty of stenosis posed on the UIA is hemodynamic changes, which is usually increased blood and pressure. Whether there are more chances of UIA rupture is upsetting. The result of this study showed that simultaneously UIA embolization with non-adjacent stenosis angioplasty may be performed without additional ischemic stroke risks. For separate session patients, there is also no hemorrhagic stroke during a short interval, indicating that staged management is also optional.

The reported overall complications rate of endovascular treatment for intracranial stenosis is around 10%, and the unfavorable clinical outcome rate is around 5% (20–22). The reported complications rate of endovascular treatment for UIAs is 4.9% (23) and unfavorable outcome is around 4.8% (24). A total of 3 ischemic complications happened in this cohort. The complication incidence in this cohort is 13.6% of patients and 8.8% of procedures. Clinical follow-up revealed no ischemic and hemorrhagic stroke and all patients achieved favorable clinical outcome, indicating that the outcome of endovascular treatment for non-adjacent incidental UIA associated with symptomatic intracranial stenosis was relatively good.

### Limitations of the study

Our study has several limitations. There is no statistical significance between one session group and separate session groups. This may due to small sample size and low complication incidence. Moreover, this is a retrospective study instead of randomized trial. More patients with ipsilateral stenosis and aneurysm are inclined to be treated at the same session. This may conceal some selection bias such as potential ischemic complication risks or UIA rupture risk. The result of the study needs to be confirmed by large cohort.

## Conclusions

Non-adjacent concomitant UIA and severe symptomatic intracranial atherosclerotic stenosis will not pose additional endovascular treatment risks. Both the simultaneous endovascular management and short intervals between separated procedures are technically feasible and safe.

## Data availability statement

The original contributions presented in the study are included in the article/supplementary material, further inquiries can be directed to the corresponding author/s.

## Ethics statement

The studies involving human participants were reviewed and approved by Medical Ethics Committee of Beijing Tiantan Hospital. The patients/participants provided their written informed consent to participate in this study.

## Author contributions

YL and HH had substantial contributions to conception, design of the study, and final approval of the version to be published. XM and JW had main responsibility for experiment, acquisition of data, and data analysis. HJ write the article. All authors have read and approved the final manuscript.

## Funding

This study was supported by the Beijing Gold-Bridge Project (Grant Number ZZ21060) and the National Natural Science Foundation of China (Grant Number 82171289).

## Conflict of interest

The authors declare that the research was conducted in the absence of any commercial or financial relationships that could be construed as a potential conflict of interest. The reviewer TW declared a shared parent affiliation with the authors HJ, HH, and YL to the handling editor at the time of review.

## Publisher's note

All claims expressed in this article are solely those of the authors and do not necessarily represent those of their affiliated organizations, or those of the publisher, the editors and the reviewers. Any product that may be evaluated in this article, or claim that may be made by its manufacturer, is not guaranteed or endorsed by the publisher.
